# Supplementary figures and images for: Effect of seed hydro-priming durations on germination and seedling growth of bitter gourd (Momordica charantia)
Source: PLoS One. 2021 Aug 5;16(8):e0255258. doi: 10.1371/journal.pone.0255258 (PMC8341585; doi:10.1371/journal.pone.0255258)

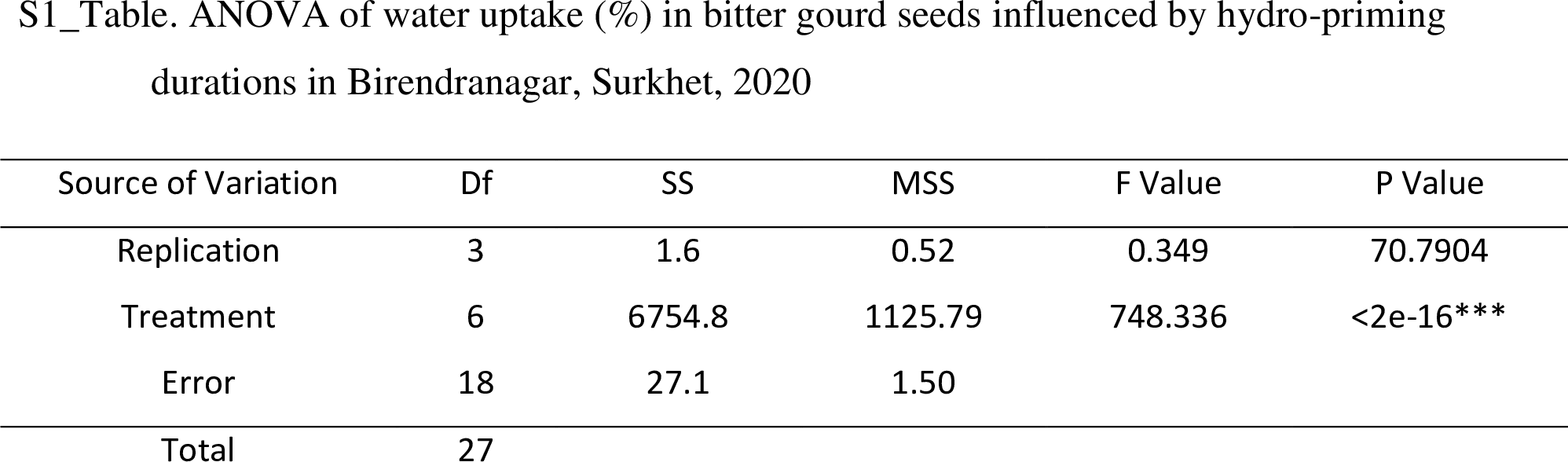

Supplement: S1 Table — (TIF) [file pone.0255258.s001.tif]

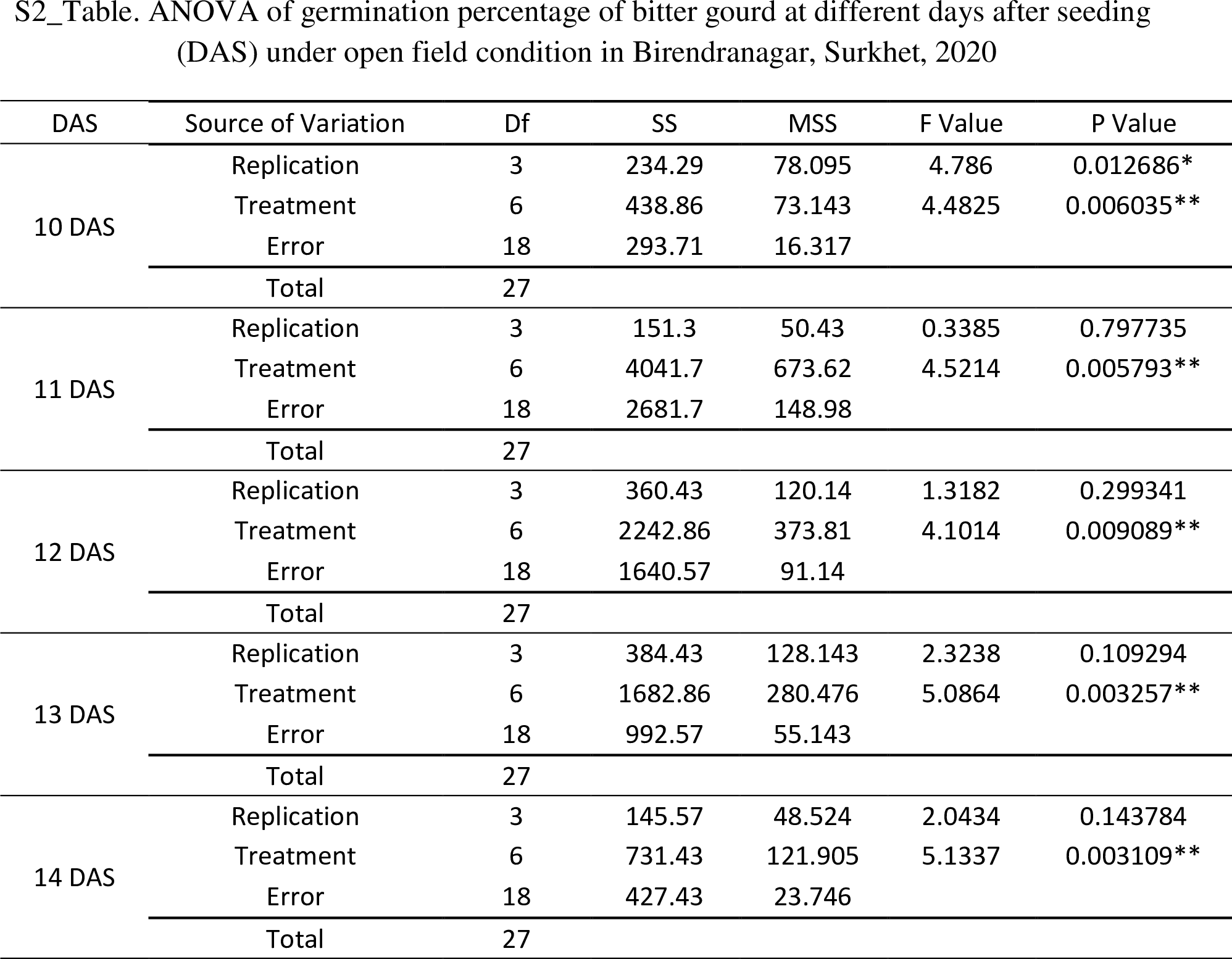

Supplement: S2 Table — (TIF) [file pone.0255258.s002.tif]

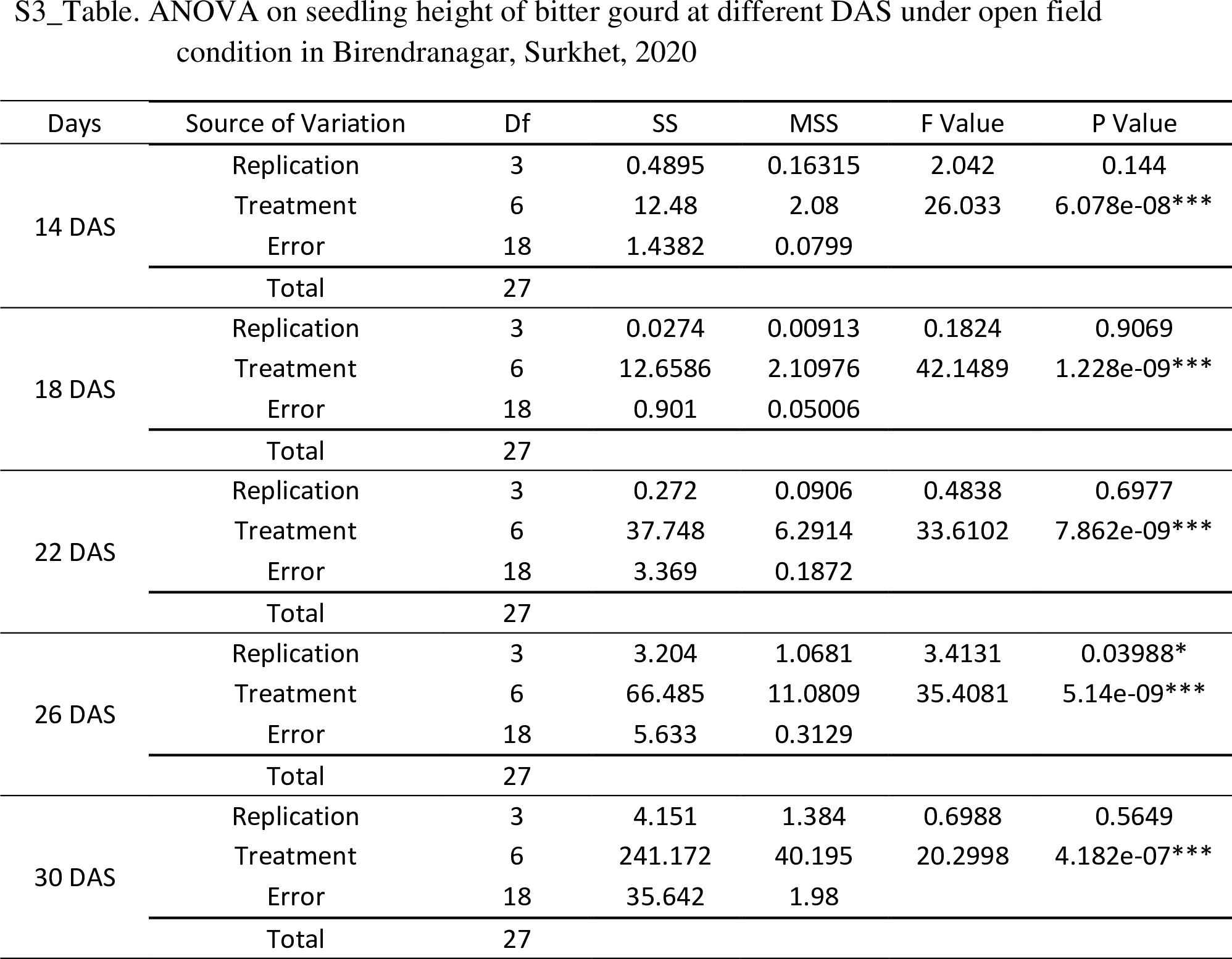

Supplement: S3 Table — (TIF) [file pone.0255258.s003.tif]

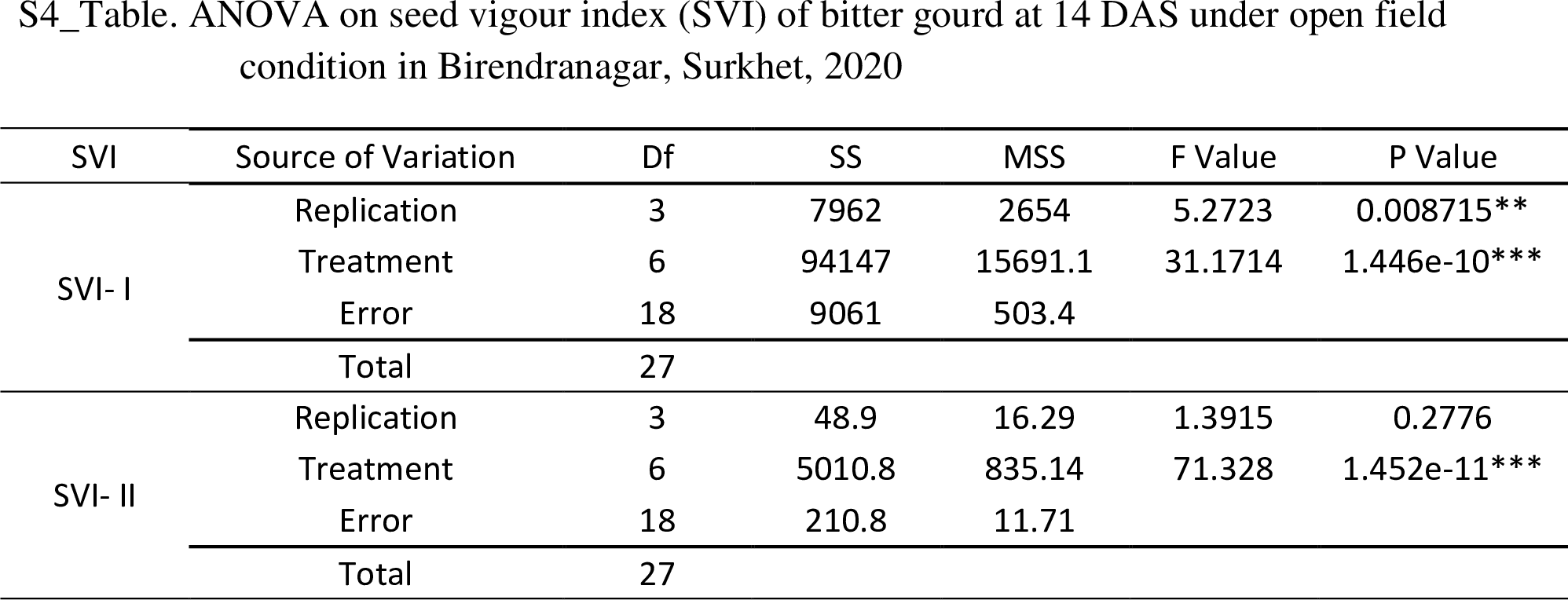

Supplement: S4 Table — (TIF) [file pone.0255258.s004.tif]
