# Supplementary material for: Effect of seed hydro-priming durations on germination and seedling growth of bitter gourd (Momordica charantia)
Source: PLoS One. 2021 Aug 5;16(8):e0255258. doi: 10.1371/journal.pone.0255258 (PMC8341585; doi:10.1371/journal.pone.0255258)
Supplement: Attachment — Submitted filename: Comments_Saidur_D-21-08775.docx [file pone.0255258.s005.docx]

**Comments**

The authors found in the experiment, seed hydro-priming was found to be effective for increasing germination and seedling growth in bitter gourd only on the basis of some morphological parameters. However, there is a space to add more morphological parameters to conclude the results. For publication in such prestigious journal, I recommend major revisions.

Line 21. In abstract Please mention all the priming durations otherwise abstract will not be self-explanatory. Before going to results its need to specify the methods adopted in the study.

Line 36. The crop is of high economic and nutritional importance. Please rewrite the sentence.

The introduction is poorly citied. For instance, in case of hydro priming only one citation is written. However, recently many work has been conducting on priming and hydro-priming. Please cite more recent reference and I think also space for writing some introductory notes on priming before hydro-priming.

Authors should more careful on grammatical mistakes in the introduction.

Line 91-92 Authors mentioned no significant difference was found after 36 hours of hydro-priming as shown in Fig 1. But in figure its looks not clear because there is no sign of statistical analysis.

The experiments lack of some data such as **minerals homeostasis uptake data**, **water related traits,** can be checked because author discussed priming can be attributed to an increased nutrient usage capacity that allows for a higher relative growth rate and better plant water status regulation. Also, **many others morphological parameters** can be added.

In the discussion, results did not discusses properly authors only focus the literature. Discussion section should be more clarified with discussions and results.

Overall, the writing lack of many references, more literature should be added. On the other hand, **climatic condition in the experiment** time was an important factor. However, there is no information about climatic condition of the experimental field. **Moreover, one trial in the field for one year is not sufficient to conclude, it needs at least 2-3 trial on 2-3 consecutive years**.
