# Supplementary material for: Effect of seed hydro-priming durations on germination and seedling growth of bitter gourd (Momordica charantia)
Source: PLoS One. 2021 Aug 5;16(8):e0255258. doi: 10.1371/journal.pone.0255258 (PMC8341585; doi:10.1371/journal.pone.0255258)
Supplement: Attachment — Submitted filename: Response to Reviewers.docx [file pone.0255258.s006.docx]

Manuscript PONE-D-21-08738

10^th^ June 2020

Dear Editors

PLOS ONE

We express our gratitude for the valuable and constructive review of our manuscript. The provided suggestions and comments have helped us to further strengthen the overall quality of the paper. With utmost care, we have incorporated the requested suggestions/corrections as proposed by the reviewers. The specific responses to the reviewer’s comments, in blue, are listed below. All page numbers refer to the revised manuscript file.

Reviewer #1: Comments

- Line 21. In abstract Please mention all the priming durations otherwise abstract will not be self-explanatory. Before going to results its need to specify the methods adopted in the study.

Thank you for pointing this out. We have included the priming durations.

- Line 36. The crop is of high economic and nutritional importance. Please rewrite the sentence.

As suggested by reviewer, we have revised the sentence. The revised sentence reads as “The crop is of high nutritional and medicinal importance. Its immature fruit is rich source of dietary fibers, minerals and Vitamins (C and A) which also acts as a blood purifier and is highly beneficial to diabetes patients. Likewise, it also has anti-carcinogenic property and can be used against multiple cancer forms as a cytostatic agent”.

- The introduction is poorly citied. For instance, in case of hydro priming only one citation is written. However, recently many work has been conducting on priming and hydro-priming. Please cite more recent reference and I think also space for writing some introductory notes on priming before hydro-priming.

We have added the suggested content to the manuscript (line 49-62).

- Authors should more careful on grammatical mistakes in the introduction.

Thank you for your suggestions. We have worked out on this and corrected the errors.

- Line 91-92 Authors mentioned no significant difference was found after 36 hours of hydro-priming as shown in Fig 1. But in figure its looks not clear because there is no sign of statistical analysis.

Thank you for pointing this out. We have added the respective table with necessary sign of statistical analysis (Table 1 and 2).

- In the discussion, results did not discusses properly authors only focus the literature. Discussion section should be more clarified with discussions and results.

A suggested by the reviewer, we have clarified discussion section with discussions and results.

- The experiments lack of some data such as minerals homeostasis uptake data, water related traits, can be checked because author discussed priming can be attributed to an increased nutrient usage capacity that allows for a higher relative growth rate and better plant water status regulation. Also, many others morphological parameters can be added.

Thank you for pointing this out. We have taken data related to root length and seedling dry weight too along with water imbibition, germination percentage and seedling height. Since, they are the major factor in calculating seedling vigour index I and II, we haven’t included them separately.

- Overall, the writing lack of many references, more literature should be added.

As per suggestions, we have read more literatures and included in the manuscript.

- On the other hand, climatic condition in the experiment time was an important factor. However, there is no information about climatic condition of the experimental field.

We think this is the vital suggestion. We have included climatic details in the line 79-87.

- Moreover, one trial in the field for one year is not sufficient to conclude, it needs at least 2-3 trial on 2-3 consecutive years.

Reviewer #2:

- Give reference for the statement given on line number 43-44.
- Give reference for this statement given on line 172-174.

Thank you for pointing this out. We have added the references for respective sentences in given line numbers.

- Please provide experimental site details i.e. location, soil, weather etc at the beginning of materials and methods.

We think this is an important suggestion. We have included those details on line number 79-87.

- Mention source of the seed, its initial germination percentage and class of the seed at line number 67.

As per suggestions we have included the detail on line number 90 and 91.

- The formula given on line number 71 seems wrong. It should be weight of seed after priming - weight of seed before priming/weight of seed before priming.

The formula is corrected as per the reviewer’s suggestion.

- A reference of Table 7 has been given on line number 130 however there is no Table 7 in the whole text.

Thank you for pointing this out. We have now added the tables (Table 1 and 2) to for clarification of the reference.

- Why don’t you recommend 36 hours seed priming instead of 48 hours which has similar results in most cases?

We agree with the reviewer’s suggestion. Accordingly, we have revised our conclusion to recommend 36 hours of hydro-priming for bitter gourd seed prior to seeding/sowing.

- Most of the references are old which can be replaced by latest ones.

As per the suggestions, we have cited more recent literatures.

We look forward to hearing from you regarding our submission. We would be glad to respond to any further questions and comments that you may have.

Kind regards

Binod Adhikari
